# Development of a Climate Change Vulnerability Assessment Using a Public Health Lens to Determine Local Health Vulnerabilities: An Ontario Health Unit Experience

**DOI:** 10.3390/ijerph15102237

**Published:** 2018-10-12

**Authors:** Morgan M. Levison, Ainslie J. Butler, Steven Rebellato, Brenda Armstrong, Marina Whelan, Charles Gardner

**Affiliations:** Simcoe Muskoka District Health Unit, Barrie, ON L4M 6K9, Canada; ainslie.butler@smdhu.org (A.J.B.); steven.rebellato@smdhu.org (S.R.); brenda.armstrong@smdhu.org (B.A.); marina.whelan13@gmail.com (M.W.); charles.gardner@smdhu.org (C.G.)

**Keywords:** climate change, vulnerability, public health, adaptation, assessment, health impacts, climate change and health, adaptive capacity, climate models

## Abstract

Climate change is negatively impacting the health of Canadians and is accordingly expected to have a significant impact on public health agencies and their response to these health impacts throughout the twenty-first century. While national and international research and assessments have explored the potential human health impacts of climate change, few assessments have explored the implications of climate change from a local public health perspective. An applied research approach to expand local knowledge and action of health vulnerabilities through a climate change action plan and vulnerability assessment was utilized by a local public health agency. Adoption and adaptation of the approach used may be valuable for public health organizations to assist their communities. Through completing a vulnerability assessment, an evidentiary base was generated for public health to inform adaptation actions to reduce negative health impacts and increase resiliency. Challenges in completing vulnerability assessments at the local level include the framing and scoping of health impacts and associated indicators, as well as access to internal expertise surrounding the analysis of data. While access to quantitative data may be limiting at the local level, qualitative data can enhance knowledge of local impacts, while also supporting the creation of key partnerships with community stakeholders which can ensure climate action continues beyond the scope of the vulnerability assessment.

## 1. Introduction

Climate change is negatively impacting the health of Canadians and is accordingly expected to have a significant impact on public health throughout the twenty-first century [1,2]. Climate-sensitive health vulnerabilities, including issues of extreme temperatures, air quality, food- and water-borne illness and security, and vector-borne diseases exist across Canada [2,3,4]. Impacts due to climate change can be both direct (e.g., health effects of air pollution and temperature stress; increased range and transmission of infectious diseases; reduced access to safe water; injuries related to extreme weather events) and indirect (e.g., food insecurity; psychosocial impacts; population displacement) [5,6,7]. The health outcomes of climate change can manifest at global, national, provincial, and local levels. While national and international research and assessments have explored the potential impacts of climate change on human health at global and national levels [2,4,8,9,10,11,12], few assessments have explored the implications of climate change from a local public health perspective, especially within the Canadian context [13,14,15,16,17].

Public health units in Ontario, Canada are provincially and locally funded health agencies established to provide efficient community health programs. They are mandated to protect and promote health on a community scale and therefore need evidence to increase understanding of the local context of climate change [18,19]. In addition to traditional preventative health programs, local public health units are well positioned to consider local health status and determinants of health when assessing populations vulnerable to climate change and potential adaptive capacity measures [20,21,22]. This perspective is imperative to decrease negative climate-sensitive health outcomes, as effective adaptation to climate change is a complex issue that will require drivers from multiple levels of government [23,24].

Public health professionals are increasingly engaged in identifying public health vulnerabilities due to climate change, and provincial public health mandates may require the consideration of climate change mitigation and adaptation by local public health authorities [18,25]. Within Ontario, a focus on climate change programming and planning in public health is emphasized through the inclusion of climate change within the updated 2018 Ontario Public Health Standards [18,19]. In addition, supporting resources, such as the Ontario Climate Change and Health Vulnerability and Adaptation Assessment Toolkit have been created to assist local public health units with critically examining current and future climate change impacts, vulnerable populations and adaptive capacity measures to increase resiliency to associated challenges [26,27,28].

This manuscript describes the process of conducting a vulnerability assessment at a local public health unit level. It describes the challenges faced, lessons learned, and identifies the utility of a climate change and health vulnerability assessment to support climate planning and community collaboration surrounding climate change and health within a local public health context.

### 1.1. Identifying Climate Change as a Priority Public Health Issue

Public health units within Ontario must continually adapt to and address changing health needs within a community. As such, the Simcoe Muskoka District Health Unit (SMDHU) identified the ability to identify a priority public health issue which required a coordinated and comprehensive agency response as a key component of the 2012–2016 strategic plan. Key factors such as support from the Medical Officer of Health and an internal recognition of climate change as an issue which spans all public health departments and programs provided the impetus for submitting climate change for consideration within the priority public health issue process.

As part of the selection process, a situational assessment was conducted which reviewed the local need to address climate change, through examining historical climate data, potential health impacts related to climate change, and current gaps within knowledge of climate-health risks. This situational assessment illustrated the expanding need to support the health of Simcoe Muskoka residents in the face of climate change. As such, in 2014, the SMDHU, with support of its Board of Health, identified climate change as the priority public health issue for the health unit.

Once selected as the priority issue for the agency, a Climate Change Action Plan (CCAP) was developed to guide the actions taken and resources required to address health impacts from climate change within Simcoe Muskoka. The CCAP also identified the required governance support and dedicated resources to allow for long-term planning. The CCAP took a three-staged approach, including: Phase I—Analysis of Current Environment; Phase II—Health Promotion and Engagement; and Phase III—the Integration of Climate Change into Health Unit Programming. Phase I involved the completion of a local climate change and health vulnerability assessment [17].

In conjunction with an impending provincial mandate to examine the health impacts of climate change, the impetus for plan development arose from a need to improve understanding of the intersection of climate change health impacts with the local health status and determinants of health, while also determining current and future climate change impacts within Simcoe Muskoka to support local mitigation and adaptation planning. The plan sought to inform agency practices and public health programming, as well as enhancing regional capacity in considering climate change health impacts.

### 1.2. Expanding Local Knowledge of Climate-Sensitive Health Vulnerabilities

Vulnerability to climate change within a public health context can be defined as the degree to which individuals or communities are prone to the health impacts of climate change [4]. Vulnerability assessments are mechanisms by which organizations can articulate current and projected impacts due to climate change. Specifically, climate change and health vulnerability assessments support increased understanding and identification of the impacts that a changing climate will have on the health of a population [12,29]. They enhance adaptive capacity through the identification of, and informing decisions about, mitigative and adaptive measures to decrease health impacts [20,30,31,32].

Within the Simcoe Muskoka region, local environmental organizations had completed vulnerability assessments which identified projected environmental changes due to climate change [33,34]. However, a thorough examination of local health impacts due to climate-related environmental change was lacking. As an agency which utilizes evidence-informed decision making to support program planning and practice, this gap in health consideration in previous assessments further highlighted the need to complete a climate change and health vulnerability assessment as a critical first step for the SMDHU CCAP. The vulnerability assessment allowed SMDHU to explore and evaluate local available data on the health impacts of climate change and identify internal climate change mitigation and adaptation actions, including continuation or enhancement of program activities already in place. Further, the vulnerability assessment created a tool which provides information on vulnerable populations and expected climate-sensitive impacts that can support community and municipal partners in evidence-informed decision making for their climate change mitigation and adaption planning processes.

As a priority of the CCAP, the purpose of completing a local climate change and health vulnerability assessment for Simcoe Muskoka was three-fold: (1) identify current and future climate-sensitive health outcomes; (2) determine populations vulnerable to climate-sensitive health outcomes; and (3) identify policies and actions to help mitigate impacts of climate change on health.

### 1.3. The Simcoe Muskoka Region

**Figure 1:**
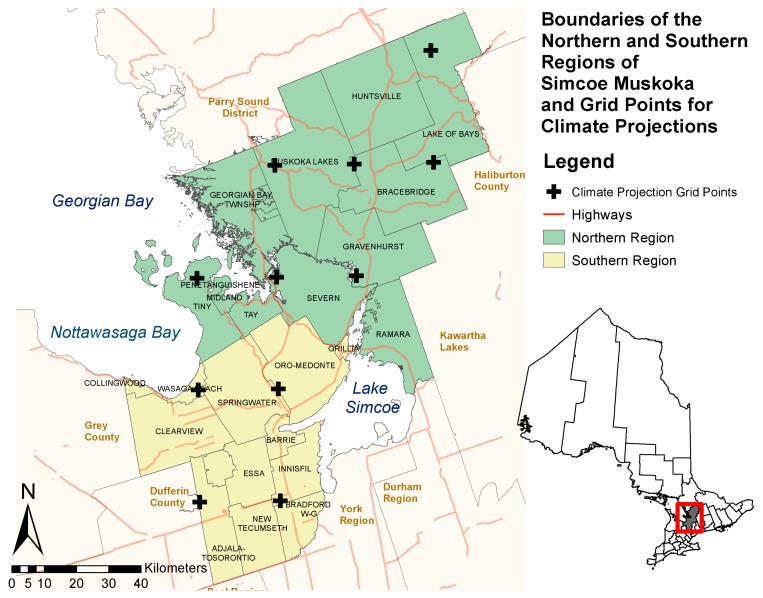
Simcoe Muskoka District Health Unit (SMDHU), Northern and Southern region boundaries and grid points for Ontario climate change portal climate projections [37], adapted from [17].

The SMDHU is responsible for health promotion and protection for residents and visitors of the County of Simcoe and the District of Muskoka, a region spanning 8,736 km^2^ in Central Ontario (Figure 1), covering 26 urban and rural municipalities. A total of 540,249 individuals reside within Simcoe Muskoka, with the majority of the population (479,650) residing within the County of Simcoe [17,35]. During summer, the population of the District of Muskoka increases by nearly 140% with the influx of tourists and summer residents [17,36]; similar estimates were not available for the County of Simcoe.

## 2. Methods

### 2.1. Assessment Framework

The SMDHU Vulnerability Assessment was completed as a primary step in the agency CCAP, which was a catalyst for and informed decisions related to climate change mitigation and adaptation. As the primary step in the assessment process, a framework which outlined the steps required for assessment completion was identified (Box 1). The assessment framework was developed from a review of international vulnerability assessment guidelines [10,12], and recently completed assessments by other Ontario public health units [13,14]. Provincial vulnerability assessment guidelines [26] were under development at the time; however, guidance was provided by the Ontario Ministry of Health and Long-Term Care based on their interim guidelines.

### 2.2. Scoping the Assessment

At the onset of an assessment, scope must be determined to define the geographical context, assess local climate-sensitive health vulnerability categories for inclusion, determine temporal considerations for climate projections, and identify required resources [10]. The scope of the SMDHU vulnerability assessment was identified in early 2016, and incorporated recently completed vulnerability assessments from Ontario public health units [13,14], resources from Health Canada [2], Natural Resources Canada [1] and the Ontario Ministry of Health and Long-Term Care [26], and consultation with internal and external stakeholders to integrate local context.

Box 1 **Actions taken to implement a Climate Change and Health Vulnerability Assessment.**

1. Formation of agency-wide steering committee
2. Vulnerability assessment creation
  - Scoping exercise with internal and external stakeholder consultation
  - Identification of key climate-sensitive health vulnerability categories
  - Identification and access to resources including internal and external data and expertise
  - Analysis and synthesis of knowledge
  - Review of draft assessment by local, provincial, and federal partners
3. Completion of a vulnerability assessment report
4. Incorporation of key results into a broader Climate Change Action Plan
5. Ongoing knowledge translation and dissemination of messages to stakeholders, community partners and other agencies to promote positive public policy change.

Adapted from: World Health Organization and Ontario Ministry of Health and Long-Term Care [10,19].

#### 2.2.1. Climate-Health Hazards and Projections

**Figure 2:**
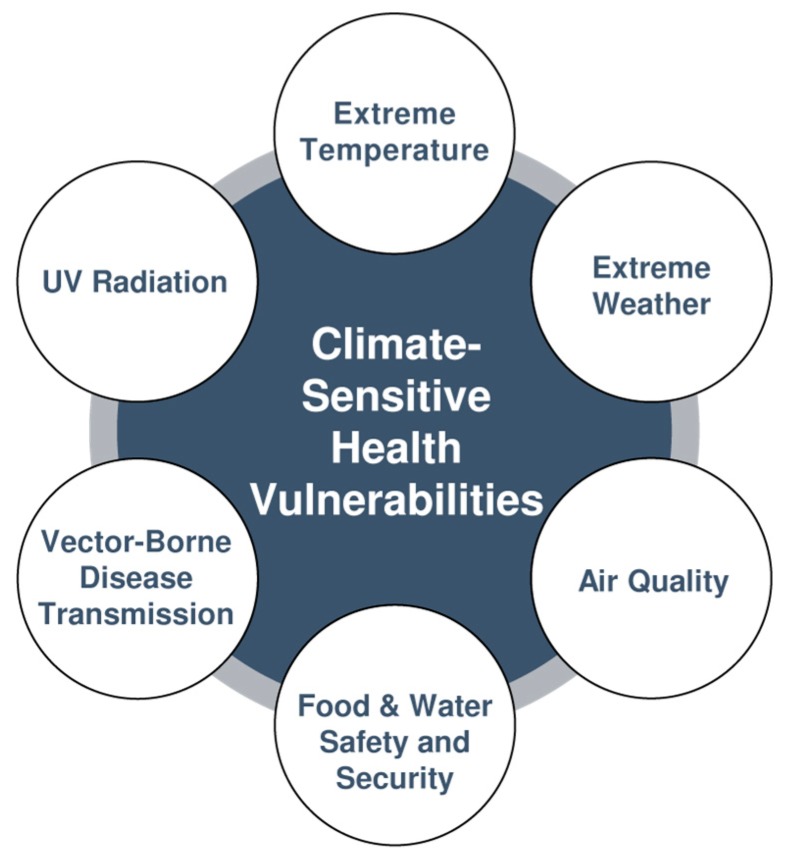
Identified climate-sensitive health vulnerability categories of importance for Simcoe Muskoka, adapted from Health Canada [2].

Six climate-sensitive health vulnerability categories of importance for Simcoe Muskoka (Figure 2) were identified from both national and international literature [1,2,4] and stakeholder consultation. Climate change projections were used to describe temperature and precipitation changes from the 1990s (baseline timeframe) to projections for the near- (2020s), short- (2050s) and long-term (2080s) futures.

#### 2.2.2. Geographical and Demographic Considerations

The SMDHU covers a wide geographic area with differing demographic, geographic, and local capacities. Accordingly, a vulnerability assessment with research relevant to the health unit as well as 26 local municipalities and local environmental and conservation authorities was required. On the basis of data availability and the location of climate projection grids (25 km × 25 km grid boxes used to subdivide the globe in climate projection modelling), the health unit was divided into two distinct geographic regions (Southern and Northern regions; Figure 1). Influences of differences in local demographics (e.g., proportion in low income, age distribution, summer residents) were analyzed both spatially and temporally.

#### 2.2.3. Resources

Decisions in the development of the vulnerability assessment were supported through an internal climate change steering committee comprised of agency staff responsible for the planning and implementation of climate change programming and staff with program expertise. Senior management, including the Medical Officer of Health, were included to ensure that the direction of the Climate Change Action Plan and vulnerability assessment were cohesive with agency strategic directions and priorities. The completion of the vulnerability assessment required direct investment of human resource capacity (with the project team including managerial, epidemiological and health promotion inputs) to support data identification, analysis, and synthesis. In addition, all program areas were engaged throughout the process to ensure a comprehensive reflection of climate change activities from an agency level. Specific program expertise for direction and interpretation was drawn upon from the social determinants of health, chronic disease prevention, communicable disease control, environmental health, and child health programs. These programs are all involved in community engagement and program delivery with activities and clients that intersect with climate change vulnerabilities.

### 2.3. Describe Current and Future Impacts

#### 2.3.1. Data Identification

Three lenses were used to describe vulnerability to climate change: exposure to climate hazards (probability of a climate-related hazard, such as an extreme weather event, occurring); sensitivity (the degree to which individual or community health is impacted by the effects of climate change); and adaptive capacity (ability of an individual or community to adapt to the changing environment) [13,26,38].

In order to describe exposures, sensitivity and potential impacts in the SMDHU population from climate-sensitive health vulnerability categories, data was required for:

- current and projected local climate (i.e., temperature and precipitation);
- current and projected population distributions;
- current and historic prevalence of climate-sensitive health outcomes; and
- prevalence of behaviors that modify the sensitivity to these climate sensitive health outcomes (e.g., sun safety behaviors).

Where required data such as projected precipitation data or flood plain maps were not available to the health unit, local, regional, provincial and federal agencies were contacted to request access and support.

#### 2.3.2. Research in Context

Local community organizations and external experts (e.g., provincial and federal climate scientists) were engaged to review vulnerability assessment output drafts in order to ground truth the information for relevance and context.

### 2.4. Identify Current and Future Adaptive Capacity

Qualitative data collection was conducted to expand the understanding of climate change mitigation and adaption actions implemented internally and by community and municipal stakeholders. Collected data highlighted future directions and roles for climate action in public health. Semi-structured key informant interviews and focus groups were employed to achieve the research objectives.

Key informant interview and focus group protocols and materials were subject to the SMDHU Research Review process to ensure appropriate and ethical collection of data in line with the Tri-Council Policy Statement: Ethical Conduct for Research Involving Humans (TCPS 2) [39]. All participants provided informed consent.

#### 2.4.1. External Consultation

Key informant interviews were completed to identify community and municipal climate actions. Specifically, the key informant interviews aimed to: (1) increase understanding of local climate impacts and associated mitigation and adaption actions; (2) establish partners’ expectations of public health’s role in increasing community climate resiliency; and (3) identify areas for collaboration with community partners related to climate change mitigation and adaptation.

Inclusion criteria for the key informant interviews included individuals involved through employment or volunteering with climate change mitigation and adaptation activities in Simcoe or Muskoka and/or who support populations which are vulnerable to climate change. Representatives from municipalities, educational institutions, conservation authorities, environmental organizations, provincial ministries, and community-based agencies were included within the sample.

A purposeful maximum variation sampling method [40] was utilized to identify initial study participants, with snowball sampling employed to identify additional key individuals. Fifteen key informant interviews were conducted from March to October 2016. Interviews were digitally recorded with permission, and verbatim transcriptions completed. For participants not digitally recorded, manual recording of responses was completed by the primary researcher.

#### 2.4.2. Internal Consultation

Internal stakeholders were engaged to gain insight into current health unit actions which support climate change mitigation and adaptation and avenues for program enhancement to support climate action. Focus groups were used to: (1) identify potential actions/collaborations with community partners related to climate change mitigation and adaptation; (2) identify the current knowledge of staff regarding the connections between climate change and health; and (3) identify key areas for future climate action within existing health unit programs. Twenty-two focus groups were held with health unit staff, including program teams, management, Medical Officers of Health, and the Board of Health. Focus groups were facilitated through staff meetings. During the focus groups, responses were manually recorded by the primary researcher, as well as a secondary recorder.

#### 2.4.3. Qualitative Data Analysis

Analysis of transcripts and notes for the key informant interviews and focus groups was facilitated by QDA Miner Lite software version 1.4.5 (Provalis Research, Montreal, QC, Canada). Thematic codes were produced inductively and deductively, and reviewed by multiple research team members prior to analysis.

### 2.5. Communicate Results

The results of the vulnerability assessment were intended to support climate change mitigation and adaptation policy within Simcoe Muskoka. Knowledge translation and stakeholder engagement involves communicating key findings to three priority groups: health unit staff, provincial public health practitioners, and local municipal and community stakeholders.

## 3. Results

The completion of a climate change and health vulnerability assessment process resulted in the identification of local data which describes the current and future health impacts of climate change and supported the classification of climate-health vulnerabilities specific to the region. The fulsome results for local exposure, sensitivity, and adaptive capacity can be found in the SMDHU Climate Change and Health Vulnerability Assessment report [17].

### 3.1. Framing and Scoping the Assessment

A key result of the framing and scoping process of the vulnerability assessment was the identification of specific health vulnerabilities and associated indicators to examine the burden of illness for the Simcoe Muskoka region (See Table 1). These vulnerabilities were identified through national and international literature, as well as stakeholder consultation.

### 3.2. Describe Current and Future Impacts

#### 3.2.1. Data Identification

The vulnerability assessment drew on a combination of data collected by the health unit (e.g., mosquito surveillance), data routinely accessed by the health unit (e.g., census and hospitalization data) and novel data sources (e.g., climate change projections). Climate change projection data was obtained through the Ontario Climate Change Projections data portal (OCCP) hosted by York University in Ontario, Canada and supported by the Ontario Ministry of the Environment and Climate Change [41]. This projection data was critical for understanding the potential future climate of Simcoe Muskoka, and the OCCP represented a new resource for analysis of local climate projections in Ontario. See Table 2 for a list of all data sources incorporated in the vulnerability assessment.

#### 3.2.2. Climate-Health Outcomes

The assessment of vulnerability to climate change required local data associated with exposure to climate-sensitive health vulnerabilities, understanding of the local sensitivity of health outcomes to changes in climate and an understanding of the adaptive capacity of the individuals and communities in Simcoe and Muskoka. A sample of local results are presented in Box 2.

Box 2 **Select vulnerabilities due to climate change within Simcoe Muskoka. Adapted from the quantitative and qualitative results of the SMDHU Climate Change and Health Vulnerability Assessment [17].**

*Climate Projections*

- Annual mean temperatures are projected to increase by 1 °C (2020s), 3.5 °C (2050s) and 5.7 °C (2080s). This represents a shift in climate similar to Ohio (2020s), Kentucky (2050s), and Mississippi (2080s) for the Simcoe Muskoka region.
- The highest seasonal variation (up to +7.4 °C) will be observed during the autumn and winter.
- Precipitation patterns will shift, with increased precipitation during the winter and spring, and decreased precipitation during the summer. An increase in the intensity and frequency of extreme storms is projected.

*Demographics and Vulnerabilities*

- Greenfield development and densification in urban centers will support continued growth throughout Simcoe Muskoka, with a projected 42% increase from 540,249 residents in 2016 to 740,073 (2041 projection).
- The proportion of seniors is projected to increase dramatically, from 18% (99,100 individuals 65+) in 2015 to 30% (218,000 individuals 65+) by 2041.
- Approximately 12%, or 59,000 people, within Simcoe Muskoka live on low income. Higher rates of low income among children (15.6%) and adults 18–64 (12.3%) was observed.
- Between 2007 and 2014, 12% of Simcoe Muskoka households reported experiencing food insecurity at least once in the past 12 months. Food insecurity is highest among single-parent families (24%) in Simcoe Muskoka.
- Increasing growth in urban areas will increase vulnerabilities due to urban heat islands and flooding.

*Impacts to Health*

- Increases in extreme heat events will lead to an increase in heat-related hospitalizations.
- Increases in extreme weather events will lead to increased injuries, illness, and community emergencies.
- Increasing temperatures and overland flooding will increase the potential for bacteriological contamination of food and water.
- Warming temperatures will support the increased range of vectors such as those that spread West Nile virus (mosquitoes) and Lyme disease (blacklegged ticks, see Box 3*)*.

*Current and Potential Adaptive Capacity Actions*

- Implementation of extreme temperature notification systems.
- Advocating for healthy public policy related to climate change.
- Enhancing public messaging surrounding climate-related health impacts.
- Participating in municipal emergency response planning.
- Enhancing access to and preservation of green space.
- Continued surveillance of climate-related health sensitivities and burden of illness.
- Encouraging the inclusion of climate change mitigation and adaptation strategies into municipal policies and plans.

As an example of local health results that were described within the vulnerability assessment, Box 3 describes the vulnerability of Simcoe Muskoka to the issue of Lyme disease.

Box 3 **Case study examining passive tick surveillance, Lyme disease and climate change in Simcoe Muskoka.**

As a case study to illustrate the justification, burden, significance and directions identified for specific climate-sensitive health vulnerabilities, this box summarizes the information on Lyme disease presented in the SMDHU vulnerability assessment [17].

Vector-borne disease was identified as a priority climate-sensitive health outcome through consultation with internal and external stakeholders, as well as a review of national and international literature. Research has shown that the presence of disease-carrying vectors such as ticks is dependent on factors such as climate, geography, the presence of other species (e.g., reservoir hosts) and available habitats for breeding [71,72]. As the climate of Ontario changes, the distribution of habitats suitable for the proliferation of vectors, such as ticks, and the diseases that impact on human health are expected to change [71,72,73,74].

**Figure 3:**
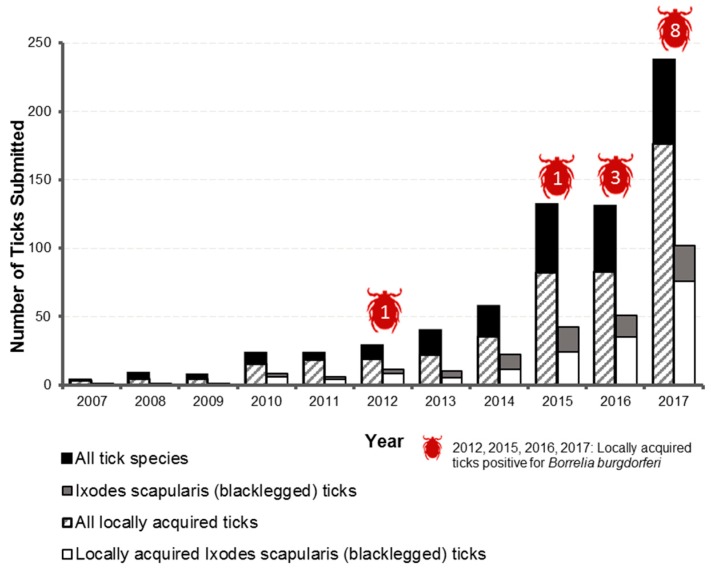
Ticks submitted to the Simcoe Muskoka District Health Unit and locally acquired ticks positive for Lyme disease, 2007–2017. Data Sources: Passive tick surveillance spreadsheet, extracted 8 May 2018. Interpretive note (1): Changes in tick submission rates are not necessarily indicative of an increase in local tick populations, and may be a result of other factors such as heightened public awareness. Interpretive note (2): Submitted ticks may originate from any location that the submitter has traveled in recent days, and not necessarily from within Simcoe Muskoka. It is estimated that two thirds of ticks submitted are acquired in Simcoe Muskoka.

Lyme disease is a disease of public health significance within the province of Ontario. To evaluate the presence of vector species, environmental surveillance is conducted. Passive surveillance for Lyme disease consists of submissions of ticks from individuals who have removed it from their (or another person’s) body and are aware the health unit can facilitate the identification and testing of ticks. Passive surveillance has indicated an increase in both the number of ticks submitted, and the number of *Ixodes scapularis* (blacklegged ticks, the main carrier of Lyme disease) identified. Figure 3 shows the increase in tick submissions from 2007 to 2017. From these submissions, over 100 blacklegged ticks have been identified and of those, thirteen tested positive for the bacteria that causes Lyme disease. The incidence of Lyme disease in humans is currently low in Simcoe Muskoka, with fewer than five cases per year on average. Provincially, the incidence rate of Lyme disease shows an increasing trend from 2000 to 2016 [17].

Research has shown clear links between climate and the distribution of Lyme disease and *Ixodes scapularis* [75]. Research suggests that the geographic range of climate-sensitive disease vectors may spread by as much as 55 km per year [1], and that by 2020, there is a strong likelihood of pervasive *I. scapularis* establishment within the health unit region [72].

Enhanced tick surveillance was identified as an adaptive capacity action for consideration, which provided supporting rationale and evidence to conduct active tick surveillance. Active surveillance for Lyme disease assists in determining the presence and abundance of *Ixodes scapularis* (blacklegged ticks), the primary vector associated with Lyme disease transmission, by collecting ticks from their natural habitat through tick dragging. The objective of active tick surveillance is to track the expansion of the habitat of blacklegged ticks and the prevalence of *Borrelia burgdorferi*. Active tick surveillance was implemented with the support of provincial and municipal partners in several areas of potential tick habitat in Simcoe Muskoka.

### 3.3. Identifying Current and Future Adaptive Capacity

Internal focus groups identified a variety of actions which currently support climate change mitigation and adaptation, such as vector-borne disease surveillance, heat notification systems, and community food security initiatives (see Box 2 above for a sample of adaptive capacity measures identified within the assessment). Focus groups identified a varying level of understanding by staff of the connection of climate change to public health and the need to incorporate climate planning into public health practice. Program areas that are already engaging in work which could be considered climate adaptive (e.g., programs supporting food security, vector-borne disease, emergency management) had an increased understanding of the linkages of climate change to current programming implementation.

Key informant interviews uncovered variations in the level of understanding of the connection to climate change and health, readiness to act on climate change mitigation and adaption, and the level of engagement throughout organizations on climate preparedness.

An additional result of the stakeholder engagement process was the creation of collaborations with community and municipal partners. Snowball sampling aided the formation of key partnerships with individuals and organizations not yet connected to the health unit in a climate change capacity. This collaboration has since evolved, leading to the creation of a regional climate change community of practice between community partners and the health unit, which will support regional action and adaption planning moving forward.

### 3.4. Communicate Results

Results were communicated internally to SMDHU staff, management, and the Board of Health, as well as externally to community partners and other public health organizations through conferences, webinars, and individual consultations.

Disseminating the results to staff increased agency awareness of local health impacts and community resiliency (e.g., climate projections, local vulnerable populations, actions to support increased resiliency). Communicating the results of the vulnerability assessment to community partners (municipalities, conservation authorities, etc.) supported enhanced collaboration with municipalities and other stakeholders. This has allowed for collaboration between the health unit and municipal staff in the creation of adaptation plans and regional climate strategies. It has also lead to the inclusion of climate change and health considerations into public policy.

By disseminating the results, as well as the process and learnings of completing a vulnerability assessment to the broader provincial public health community, communications have supported the uptake of climate actions by other public health partners.

## 4. Discussion

The SMDHU vulnerability assessment highlighted local resilience and vulnerability to climate change impacts and broadened understanding of the adaptive capacity to ongoing mitigative and adaptive programs within Simcoe and Muskoka. The process highlighted gaps in collective knowledge, resources and capacity to understand and effect change towards abating local impacts, while also highlighting the benefits of qualitative analysis and community development to support future climate action.

### 4.1. Challenges and Lessons Learned

#### 4.1.1. Framing the Assessment

Despite the support of external experts, a lack of a guidelines specific to the Ontario public health perspective was observed. While multiple vulnerability assessment guidelines existed [10,12], the availability of a context-appropriate vulnerability assessment guideline could have facilitated framework identification and assessment scope. For example, while available international guidelines identified the steps and processes for completing a vulnerability assessment, the Ontario specific guideline, released after the commencement of the SMDHU assessment, highlights indicators, relevant data sources, and adaptation considerations specific to the Ontario context [27]. Access to these indicators at the beginning of the assessment process would have supported a more systematic approach of evaluating local impacts and needs. However, by creating a framework for the local vulnerability assessment through amalgamating key strategies and actions from various vulnerability guidelines, the SMDHU assessment framework was individualized to local adaptation and mitigation needs, while also considering internal resourcing capacity.

#### 4.1.2. Scoping the Assessment

Due to the complex nature of climate change and its associated health impacts, the involvement of an agency-wide steering committee was critical for assessment completion. Inclusion of various disciplines on the steering committee supported cross-disciplinary collaboration [20], and allowed climate change to be transformed from an issue of environmental health within the agency to an issue of broader public health programming and community impact. As the completion of vulnerability assessments was not mandated at the time of the SMDHU assessment, support from senior management and the Medical Officer of Health were key in facilitating the assessment, and will be vital in supporting future action surrounding climate change [24].

Scoping the assessment provided challenges due to the geographic and demographic nature of the region. Due to the number of municipalities within the area (26), as well as the diversity of needs within each municipality (e.g., urban centers, rural farming communities, Northern tourism communities), scoping was required in order to ensure that the information examined was relevant for each community. Dividing diverse populations and geographies into discrete North and South boundaries provided the benefit of understanding the diversity of people, exposures, and health status in the population. Further, it ensured the assessment met the needs of the health unit and was reflective of the internal resourcing capacity to complete the assessment during the project timeframe.

#### 4.1.3. Describing Current and Future Risks

In the context of current and projected climate exposures, describing the local context is challenging due to the limitations in data collection (e.g., only two air quality monitoring stations in the region), and the limitations of downscaling global climate change projections to describe local changes in climate such as temperature and precipitation [12,24,76]. External experts were consulted to aid in understanding the complexity of climate change modelling and projections in the province of Ontario, which has helped to increase the reliability of climate change projections, build connections, and has led to opportunities for collaboration.

Despite increases in global climate change and public health research funding and activity [11], gaps remain in our understanding of the complex relationships between climate and health. Collaboration with research institutions and universities has been shown to be advantageous to support quantitative analysis, climate modeling and the development of vulnerability indices [20]. Due to the timing of the assessment and lack of existing partnerships with a research institution, the SDMHU vulnerability assessment was completed by internal staff. In addition, due to the rate of climate-related weather events and the size of the population within the health unit jurisdiction, some health impacts (e.g., those related to extreme weather events) were not able to be quantified. In some sectors of the vulnerability assessment, a lack of supporting research, resources and internal expertise was highlighted as a barrier to determining health projections (e.g., risk relationships and health data relating to UV exposure). The links between climate and health outcomes are complex. They can work through direct and indirect pathways and are subject to influence from other factors, including demographics [77,78]. Modelling the potential health impacts of climate change therefore becomes a wicked problem, where complex connections make projections uncertain.

Additional challenges arose from a lack of research and understanding of local sensitivity to climate-sensitive health outcomes. There are technical and logistical challenges to conducting research on outcomes at the local level [24,77], but research has shown that associations between climate and health outcomes can vary both between and within countries [79,80,81]. Research exploring the link between climate and health is limited within a North American perspective, and specifically within the Canadian or Ontario context.

#### 4.1.4. Identify Current and Future Adaptive Capacity

The integration of qualitative data collection in the vulnerability assessment process enhanced the ability to identify current and future adaptive capacity, both internally and among stakeholders. Similar to other vulnerability assessments [82], the use of qualitative data analysis supported an enhanced knowledge of community vulnerabilities and current and future adaptive capacity needs and abilities. Qualitative research methods can also act as a conduit for the identification and initiation of key community and stakeholder partnerships [20]. As an autonomous board of health responsible for engaging with 26 independent municipalities, all at varying levels of readiness to engage in climate action, the connections gained through the key informant interviews have proven invaluable in creating partnerships to support community-level action.

In addition, the completion of the focus groups with internal staff provides the opportunity to clearly examine the vulnerabilities specific to communities [22], and articulate within the assessment the breadth of work currently occurring in public health that supports adaptive capacity and enhances resiliency to climate change. While climate change can be seen as a novel aspect of public health programming and education [22], the identified actions currently in place in Simcoe and Muskoka illustrate the considerable scope of work already occurring in traditional public health programming to support climate action. This is exemplified through surveillance programs, health education and promotion activities, and other adaptive capacity measures such as warning systems and communications. Further analysis of adaptation needs and prioritization of adaptation initiatives will continue to support climate resiliency within the public health system and community as a whole.

The key informant interview process was unsuccessful in engaging local Indigenous organizations and communities due to time and resource constraints of the project. Indigenous communities face unique challenges when adapting to climate change [16]—challenges which may not be represented within the current vulnerability assessment. Future iterations of the vulnerability assessment will strive to address this gap.

#### 4.1.5. Communicating Results

Discussions and communications to staff throughout the assessment process, as well as the communication of assessment results, improved staff understanding of the linkages between climate change and public health practice. Increasing awareness of programmatic linkages is intended to support consideration of climate change adaptation or mitigation during operational planning for program delivery; this understanding is key to ensure all program areas include an integration of climate change and adaptive capacity lens during future program planning [24].

A key learning of the vulnerability assessment process was the identification of needed capacity within a health unit to better understand climate literature, models, and the need for mentorship to support enhanced evaluative skills. As evaluating community needs utilizing a climate lens is an emerging area of public health practice, public health practitioners will need to develop the skills necessary to analyze and communicate the impacts of climate change [22], a challenge which was experienced during this assessment. Communicating the results of the assessment, in addition to the process taken, challenges encountered, and lessons learned, has allowed for the strengthening of partnerships with other public health agencies, and the enhancement of public health capacity to address climate change across the province. Communicating the SMDHU vulnerability assessment experience to other local public health units can identify and mitigate key challenges, build capacity among public health professionals, and act as a framework for other public health units to learn about their own local climate context.

### 4.2. Vulnerability Assessments in the Context of Climate Change Action Plans

The climate change and health vulnerability assessment conducted by the health unit was a key first step in supporting the agency CCAP. Public health departments have a key role to play in educating policy makers on the relationship between climate change, health, and public policy [22]. Completing the CCAP afforded the ability to clearly articulate the strategy and goals of the health unit. Additionally, while communicating the impacts of climate change was indicated as an action to be completed by public health units in the Ontario public health standards, the need to complete a vulnerability assessment was not explicitly indicated at the onset of the project. Integrating the vulnerability assessment as an outcome of the larger CCAP ensured that support for enhanced human resources would be maintained by the health unit throughout the entire length of the project.

In addition, the identification of the CCAP and associated vulnerability assessment established our commitment as an agency to engage in climate action beyond the scope of the vulnerability assessment. With climate change previously primarily considered an environmental issue, the creation of action plans and vulnerability assessments provides the relevancy that public health needs to be engaged with municipalities and community organizations around the climate change table [29]. Having the scope, goals and expectations of the larger CCAP identified supports clarification of roles and management of expectations of community partners and stakeholders [22], and ensures that public health transforms from a source of health information to a partner in the community climate action process.

### 4.3. Next Steps

Results of the vulnerability assessment will be employed to support adaptation planning throughout the agency. Local impacts of climate change will vary, and thus multiple stakeholders from across the agency are critical for successful engagement to ensure adaptation actions cover the breadth of potential impacts for programs and their clients [83]. The reorientation of public health to include a climate change lens into daily practice will ensure climate change adaptation is considered during program planning for teams with relevant programmatic connections to climate change (e.g., chronic disease prevention and food security) and in the context of vulnerable populations (e.g., child health and determinants of health) [24].

In addition to internal adaptation planning, results of the vulnerability assessment will be leveraged to support stakeholder engagement and the integration of health adaptation policies among local municipalities. Local municipal governments have a key role in supporting the implementation of climate mitigation and adaptation actions which will decrease climate-sensitive health outcomes [23,84]; however, not all local municipalities and organizations have the capacity or internal resources to support local climate change vulnerability identification. The vulnerability assessment will support the health unit in giving a voice to the health impacts of climate change locally [84], and provide the evidentiary base for mitigation and adaptation strategies that decrease local climate-related health burdens.

Finally, a timeframe for a reanalysis of the vulnerability assessment will be determined based on changing demographic and community health needs.

## 5. Conclusions

The completion of a local climate change and health vulnerability assessment supported the identification of the local vulnerability and adaptive capacity to climate change. It highlighted the many activities related to climate mitigation and adaptation already occurring within public health that enhance individual and community resiliency. In addition, the assessment highlighted gaps in ability to understand the local impacts of global climate change. The applied research approach to expand local knowledge of health vulnerabilities proved valuable for public health as the evidentiary base to inform adaptation actions and program activities to reduce negative health impacts and increase resiliency. Indirect benefits of the vulnerability assessment included interdepartmental collaboration, a shifting of the paradigm away from climate change as an environmental issue to one of broader public health importance, and the opportunity to engage with community partners and stakeholders to support health adaptation across a broad range of sectors. The completion of climate change and health vulnerability assessments by Ontario public health units are important both in meeting provincial mandates and toward building an understanding of the strengths and challenges that impact local populations. Understanding local data and building partnerships with local organizations can assist in the facilitation of the creation of local policies to protect both human and environmental health.

## Figures and Tables

**Table 1:** Simcoe Muskoka climate-sensitive health vulnerabilities.

Climate-Sensitive Health Vulnerability Category	Issues of Concern	Data to Examine Burden of Illness
Extreme temperatures	Extreme heat	Heat-related illness emergency room visit rates
	Extreme cold	Hospital emergency room visits or hospitalization for cold exposure
Extreme weather	Flooding	Data unavailable
	Tornadoes	
	Forest fires	
	Winter storms	
	Drought	
Air quality	Ground-level ozone	Rates of asthma-related emergency room visits
	Particulate matter (PM_2.5_)	
	Aeroallergens	
Food and water safety and security	Food-borne illness	Monthly average cases of food- and water-borne illness
	Water-borne illness	
	Food security	
	Water security	
Vector-borne disease	Mosquito-borne illness (West Nile virus)	Incidence rate of West Nile virus cases (confirmed and probable)
	Tick-borne illness (Lyme disease)	Incidence rate of Lyme disease (confirmed and probable)
Exposure to ultraviolet radiation (UV)	Increased UV exposure	Age-specific malignant melanoma incidence rate

**Table 2:** Data types and associated sources included within the SMDHU climate change and health vulnerability assessment.

Data Type	Data Sources
Climate projections	Downscaled IPCC AR5 RCP8.5 projections [37] Local intensity-duration-frequency (IDF) curves using A1B emissions scenario [42] Projected ozone exceedances [28]
Historic climate data	Historical air quality data [43] Historical extreme weather events [44,45] Historical flood data [46,47] Historical weather data [48] Ice cover [49] Observed surface temperatures [50] Public weather alerts [51] Traffic-related air pollution [52] Wildfire risk [53]
Demographic data	2011 National Household Survey [54] 2016 Census of Population [55] Live births [56] Population estimates and projections [57,58]
Health data	Asthma prevalence [59] Canadian Community Health Survey [60] Cancer incidence, prevalence and mortality [61,62] Emergency room visits [63] Infectious diseases surveillance [64]
Risk and protective behaviors	Canadian Community Health Survey [60] Nutritious Food Basket Survey [65] Rapid Risk Factor Surveillance System [66]
Other data sources	Adverse water quality incidents [67] Beach monitoring data [68] Land use [69] Mosquito and tick surveillance [70]
